# Autistic traits in myotonic dystrophy type 1 due to MBNL inhibition and RNA mis-splicing

**DOI:** 10.21203/rs.3.rs-3221704/v1

**Published:** 2023-08-14

**Authors:** Lukasz Sznajder, Mahreen Khan, Mariam Tadross, Adam Ciesiołka, Curtis Nutter, Katarzyna Taylor, Christopher Pearson, Krzysztof Sobczak, Mark Lewis, Maurice Swanson, Ryan Yuen

**Affiliations:** University of Nevada, Las Vegas; the hospital for sick children; University of Florida; Adam Mickiewicz University; University of Florida; Adam Mickiewicz University in Poznań; Hospital for Sick Children; Adam Mickiewicz University; University of Florida; University of Florida; the hospital for sick children

**Keywords:** ASD, autism spectrum disorder, myotonic dystrophy, short tandem repeat expansion, microexon, alternative splicing, DMPK, MBNL, SRRM4, ANK2

## Abstract

Tandem repeat expansions are enriched in autism spectrum disorder, including CTG expansion in the DMPK gene that underlines myotonic muscular dystrophy type 1. Although the clinical connection of autism to myotonic dystrophy is corroborated, the molecular links remained unknown. Here, we show a mechanistic path of autism via repeat expansion in myotonic dystrophy. We found that inhibition of muscleblind-like (MBNL) splicing factors by expanded CUG RNAs alerts the splicing of autism-risk genes during brain development especially a class of autism-relevant microexons. To provide in vivo evidence that the CTG expansion and MBNL inhibition axis leads to the presentation of autistic traits, we demonstrate that CTG expansion and MBNL-null mouse models recapitulate autism-relevant mis-splicing profiles and demonstrate social deficits. Our findings indicate that DMPK CTG expansion-associated autism arises from developmental mis-splicing. Understanding this pathomechanistic connection provides an opportunity for greater in-depth investigations of mechanistic threads in autism.

Autism spectrum disorder (ASD) is a genetically and clinically heterogeneous neurodevelopmental condition that affects communication and social interactions with restricted interests and repetitive behaviors^1^. ASD affects 1 in 36 children, and more than 95% of them have at least one additional physical or mental health condition^2, 3^. Despite hundreds of genes known to confer risk for ASD, the molecular mechanisms explaining ASD and comorbid condition manifestations remain elusive^4^.

Recently, large-scale whole-genome sequencing studies identified tandem repeat mutations that contribute to ~4% of ASD risk. One the most recurrent mutations include a CTG expansion (CTG^exp^) in the 3’ untranslated region (3’UTR) of the *DMPK* gene^5, 6^. A recent large-scale newborn genetic screen estimated the prevalence of *DMPK* CTG^exp^ mutations approximately 1:2,100 newborns, with a relatively high detection rate of pre-mutations that increase the risk of germline expansions in the subsequent generation^7, 8^. The *DMPK* 3’UTR CTG^exp^ mutation causes myotonic dystrophy type 1 (DM1), one of the most variable genetic disorders with onset times that span from *in utero* to late adulthood, with highly variable symptom severity and multisystem involvement^9, 10^. Previous studies reported comorbidity of DM1 and ASD, and showed that the presence of ASD inversely correlates with DM1 age of onset^11–15^. However, a molecular mechanism explaining the manifestation of ASD in DM1 families is unknown.

In DM1 neurons and muscles, DMPK 3’UTR CUG^exp^ RNAs provide a large array of high-affinity binding sites for muscleblind-like (MBNL) RNA-binding proteins resulting in MBNL inhibition and formation of biomolecular condensates known as RNA foci^16–22^. MBNL proteins, including MBNL1 and MBNL2, are *trans*-acting factors regulating alternative splicing (AS) during organism development^19, 23^. MBNL inhibition leads to adult-to-fetal reversion of the AS program resulting in specific DM1 clinical symptoms, including myotonia^24–26^.

Developmental mis-splicing is not only a feature of DM1 but also ASD^27–29^. Neuronal microexons (miEs), which are 3 to ~30 nucleotides (nt) in length, are estimated to be mis-regulated in one-third of idiopathic ASD brains^30^. MiEs play an essential role in nervous system development and function by encoding post-translational modification sites and modulating protein-protein interaction networks^31, 32^. Importantly, alterations that recapitulate neuronal miE mis-splicing can lead to ASD-like phenotypes in mice, including social avoidance^33, 34^. Although numerous DM1 mouse models have been utilized to investigate typical DM1 manifestations, the ASD traits have not been elucidated^35, 36^. A molecular mechanism explaining the manifestation of ASD in DM1 families could open both diagnostic and therapeutic avenues.

Here, we performed an integrative transcriptomic and genomic analysis of RNA mis-splicing in human DM1 and ASD brains as well as multiple DM1 mouse models. For greater in-depth analysis, we focused on the mis-splicing of neuronal miEs in high-confidence ASD-risk genes directly regulated by MBNL proteins during human and mouse brain development. Finally, we assessed social interaction deficits in specific DM1 mouse models, a *Dmpk* 3’UTR CTG^exp^ knock-in (KI), as well as a *Mbnl* knock-out (KO) mouse models. Our results provide insights into the molecular mechanism underlying DM1-associated ASD where developmental mis-splicing of ASD-linked genes arises by loss of MBNL activity due to CUG repeat expansions.

## Results

### ASD-risk gene mis-splicing in human DM1 prefrontal cortex

**Figure 1:**
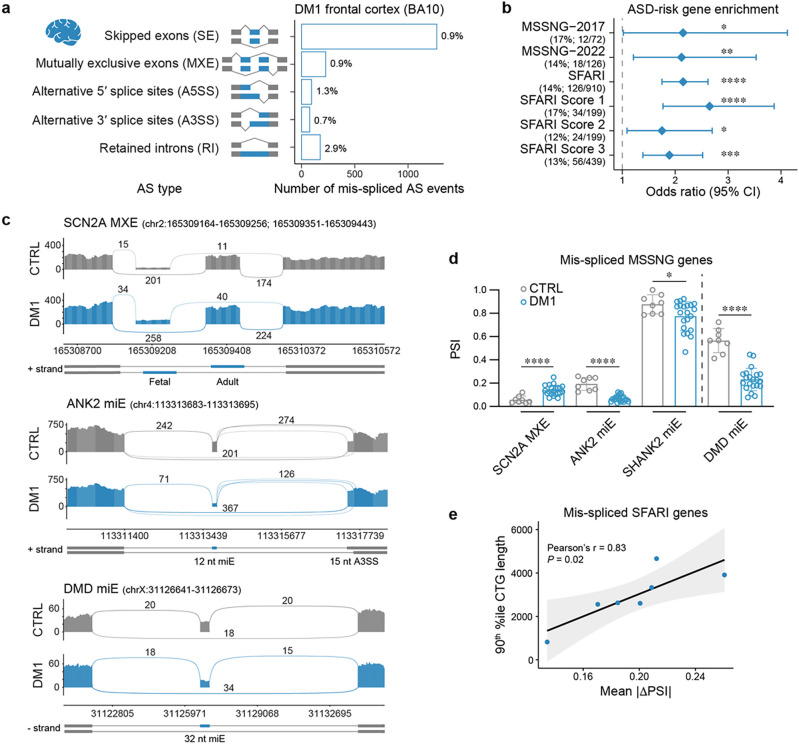
ASD-risk gene mis-splicing in DM1 prefrontal cortex. **a**, Differential AS analysis in DM1 (N = 21: N_XX_ = 12, N_XY_ = 9; sampling age: median = 56 years (y), min = 39y, max = 77y; unknown ASD status) compared to age-matched control (CTRL; N = 8: N_XX_ = 4, N_XY_ = 4; sampling age: median = 63y, min = 48y, max = 71y) prefrontal cortex (Brodmann area 10; BA10) RNA-seq samples. AS mis-splicing criteria: |DPSI | > 0.1, FDR < 0.05. The bar graph shows the number and percentage of significantly mis-spliced AS event types. **b**, MSSNG-2017, MSSNG-2022 and SFARI gene-set enrichment analysis for mis-spliced genes in DM1 BA10. Points represent the odds ratio (OR), and error bars represent the 95% confidence interval (CI). The vertical dashed line represents OR = 1. **c**, Sashimi plot of DM1 (N = 8) and CTRL (N = 8) BA10 RNA-seq samples for SCN2A MXE, ANK2 miE, and DMD miE. **d**, SCN2A MXE, ANK2 miE, SHANK2 miE, and DMD miE (only MSSNG-2017) mis-splicing in DM1 BA10. The bar graph shows mean percent spliced-in (PSI) ± standard deviation (SD). **e**, Correlation between previously estimated 90^th^ percentile of CTG repeat lengths (N = 7)^38^ and mean |DPSI| values for mis-spliced SFARI genes in DM1 BA10. **b,d**, * FDR < 0.05, ** FDR < 0.01, *** FDR < 0.001, **** FDR < 0.0001.

The prefrontal cortex orchestrates executive functions affected in ASD, and previous studies reported transcriptome-wide changes in this brain region^29, 37^. To test the hypothesis that the *DMPK* 3’UTR CTG^exp^ mutation leads to mis-splicing of ASD-risk genes, we analyzed human prefrontal cortex (Brodmann area 10; BA10) RNA-seq data generated from DM1 (unknown ASD status) and unaffected control samples (Supplementary Table 1)^38^. For differential AS analysis, we computed the change of percent spliced in (DPSI) for skipped exons (SE), mutually exclusive exons (MXE), alternative 5′ and 3′ splice sites (A5SS and A3SS) and retained introns (RI). Of all identified AS events (100%) and genes (100%) in DM1 cortex splicing analysis, 1% of AS events met our mis-splicing criteria in the total pool of 7% of mis-spliced genes (Fig. 1a and Supplementary Fig. 1a). To investigate the DM1 splicing profile in genes related to ASD, we retrieved 38 ASD-relevant gene sets from previous studies and available databases (Supplementary Table 2). Our statistical analysis revealed a significant enrichment of mis-spliced events for 76% of the gene sets (Supplementary Fig. 1b). Importantly, there was a significant enrichment of genes from the Simons Foundation Autism Research Initiative (SFARI; OR = 2.2, FDR = 1.6 × 10^−11^) database (Fig. 1b and Supplementary Fig. 1b), including SFARI’s ‘high confidence’ (Score 1; OR = 2.7, FDR = 7.3 × 10^−6^), ‘strong candidate’ (Score 2; OR = 1.8, FDR = 2.7 × 10^−2^) and ‘suggestive evidence’ (Score 3; OR = 1.9, FDR = 1.1 × 10^−4^) gene categories. Our analysis also revealed a significant enrichment of high-confidence ASD-risk genes identified in two large Autism Speaks MSSNG-based whole-genome sequencing studies: MSSNG-2017^39^ (OR = 2.2 , FDR = 3.4 × 10^−2^) and MSSNG-2022^40^ (OR = 2.1, FDR = 8.3 × 10^−3^) (Fig. 1b and Supplementary Fig. 1b). Out of 36 overlapping ASD-risk genes in both MSSNG-2017 and MSSNG-2022 studies, 17% were mis-spliced in DM1 cortex, including *SCN2A, ANK2, SHANK2*. We also identified mis-splicing in the *DMD* gene (Fig. 1c–d and Supplementary Fig. 1c). Mutations in DMD mediate Duchenne muscular dystrophy which can be comorbid with ASD^41, 42^.

To test whether the level of ASD-risk gene mis-splicing was associated with the degree of CTG^exp^ in DM1 prefrontal cortex, we correlated the CTG repeat length with the mean |DPSI | values for the mis-spliced ASD-risk genes in DM1. We selected previously determined repeat sizes corresponding to the 90^th^ percentile of CTG length distribution (Supplementary Table 1) since a previous study demonstrated the strongest positive correlation between those CTG sizes and general mis-splicing level in DM1 prefrontal cortexes^38^. This analysis revealed a significant positive correlation between CTG^exp^ size and the number of mis-spliced events in ASD-risk genes from the SFARI (*r* = 0.83, *P* = 0.02) and MSSNG-2017 study (*r* = 0.81, *P* = 0.03) (Fig. 1e and Supplementary Fig. 1d). Collectively, these results indicated that the *DMPK* 3’UTR CTG^exp^ mutation in the prefrontal cortex perturbs the splicing of ASD-relevant genes.

### Microexon mis-splicing of ASD-risk genes in mouse *Mbnl1*; *Mbnl2* conditional double knockout frontal cortex

**Figure 2:**
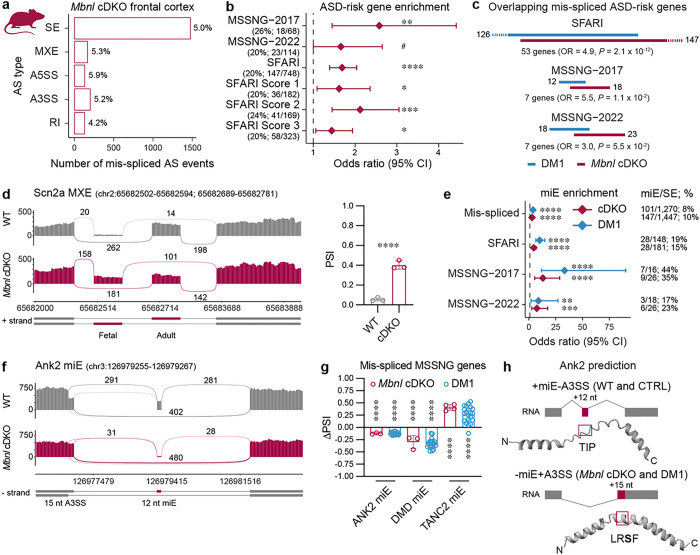
Microexon mis-splicing in DM1 and *Mbnl* cDKO frontal cortexes. **a**, Differential AS analysis in *Mbnl* cDKO (N_XY_ = 3) compared to littermate WT control (N_XY_ = 3) frontal cortex RNA-seq samples. The bar graph shows the number and percentage of significantly mis-spliced AS event types (|DPSI | > 0.1, FDR < 0.05). **b**, MSSNG-2017, MSSNG-2022 and SFARI gene-set enrichment analysis for mis-spliced genes in *Mbnl* cDKO frontal cortex. Points represent the OR and error bars represent the 95% CI. The vertical dashed line represents OR = 1. **c**, Overlap between mis-spliced SFARI, MSSNG-2017 and MSSNG-2022 genes in DM1 and *Mbnl* cDKO cortexes. **d**, Scn2a MXE mis-splicing. Sashimi plot of *Mbnl* cDKO and WT RNA-seq samples. The bar graph shows the mean PSI ± SD. **e**, miE enrichment analysis for SFARI, MSSNG-2017 and MSSNG-2022 mis-spliced SE events. **f**, Sashimi plot of *Mbnl* cDKO and WT RNA-seq samples for Ank2 miE 12 nt. **g**, Human and mouse miE mis-splicing in DM1 and *Mbnl* cDKO. The bar graph shows mean DPSI ± SD. **h**, Schematic of Ank2 miE to the A3SS coordinate splicing and modeled structures of mouse Ank2 polypeptides. The aa sequences changed by AS are by a magenta box. The S901 phosphorylation site is bolded. **b,d,e,g**, # FDR = 0.056, * FDR < 0.05, ** FDR < 0.01, *** FDR < 0.001, **** FDR < 0.0001.

The strong correlation between CTG^exp^ length and the degree of ASD-risk gene transcript mis-splicing suggests the involvement of MBNL regulation in the prefrontal cortex. To test this possibility, we performed differential AS analysis on RNA-seq data from adult *Mbnl1*^−/−^; *Mbnl2*^*c*/c^; Nestin-*Cre*^+/−^ conditional double knockout mice (*Mbnl1; Mbnl2* cDKO, hereafter *Mbnl* cDKO) versus wild-type (WT) frontal cortex samples^43^. The *Mbnl* cDKO mice bypass the embryonic lethality of constitutive *Mbnl1*^−/−^; *Mbnl2*^−/−^ DKO mice and provide a nervous system-specific model where *Mbnl1* expression is absent in all tissues while *Mbnl2* is lost only in neuronal and glial precursor cells. The *Mbnl* cDKO is characterized by RNA mis-splicing, altered cortical neuronal and synaptic structures and widespread brain anatomical changes^43–46^. In total, 5% of AS events in 13% of detected genes were mis-spliced (Fig. 2a and Supplementary Fig. 2a). Similar to human DM1, 61% of ASD-relevant gene lists were significantly enriched among the mis-spliced genes in the *Mbnl* cDKO frontal cortex, including ASD-risk genes from the SFARI (OR = 1.7, FDR = 1.2 × 10^−6^), MSSNG-2017(OR = 2.6, FDR = 2.6 × 10^−3^) and MSSNG-2022 (OR = 1.7, FDR = 5.6 × 10^−2^) studies (Fig. 2b and Supplementary Fig. 2b). The overlap between SFARI, MSSNG-2017 and MSSNG-2022 mis-spliced ASD-risk genes in DM1 and *Mbnl* cDKO frontal cortexes varied from 30% to 58% (Fig. 2c). In total, we identified a significant overlap of 55 mis-spliced ASD-risk genes (e.g., *SCN2A*) between mouse and human frontal cortex (OR = 1.7, *P* = 1.6 × 10^−12^, Fisher’s exact test) (Fig. 2d).

Neuronal miE (defined here as a 3–33 bp SE) mis-splicing is a hallmark of ASD brains, which can lead to ASD-like behaviors in mice^30, 33, 34^. We noticed significant disproportional miE mis-spliced events in both *Mbnl* cDKO and DM1 cortex (Fig. 2e). MiEs constituted 4% of all detected SE events in WT mouse frontal cortex but represented 10% ofmis-spliced SE events in *Mbnl* cDKO (OR = 2.9, FDR = 2.4 × 10^−24^), as well as 15%, 23% and 35% when they are found within ASD-risk genes from the SFARI (OR = 4.5, FDR = 1.5 × 10^−9^), MSSNG-2022 (OR = 7.4, FDR = 4.5 × 10^−4^), and MSSNG-2017 (OR = 13.0, FDR = 5.4 × 10^−7^) studies, respectively. Similarly, the proportion of miE mis-splicing events increased from 2% to 8% in DM1 prefrontal cortex (OR = 3.8, FDR = 8.1 × 10^−26^), and to 19%, 17%, and 44% in ASD-risk genes from the SFARI (OR = 10.0, FDR = 2.2 × 10^−17^), MSSNG-2022 (OR = 8.8, FDR = 7.7 × 10^−3^), and MSSNG-2017 (OR = 33.2, FDR = 4.4 × 10^−8^) studies, respectively. In total, we identified mis-spliced miE events in 33 genes that are present in both human DM1 and mouse *Mbnl* cDKO cortex, including evolutionary conserved miEs in high-confidence ASD-risk genes, such as *ANK2, TANC2*, and *DMD* (Fig. 2f–g).

Since previous studies have shown that miEs can locally modulate protein structure^30^, we performed comparative *in silico* modeling of peptides with/without miE-encoded amino acid (aa) sequences to test their potential for protein modulation. This analysis showed that some mis-spliced miEs might modulate internal (*e.g*., Ank2 and Nrxn1) or C-terminal (*e.g*., Dmd and Shank3) protein structures (Fig. 2h and Supplementary Fig. 2c-d). For example, the inclusionof thehighly conserved *Ank2* miE (12 nt) along with the use of a proximal alternative 3′ splice sites (A3SS) results in protein isoform with a TIP aa sequence, whereas miE exclusion promotes distal A3SS usage (15 nt), and results in a protein isoform with a LRSF aa sequence containing a S901 phosphorylation site^47^ (Fig. 2h and Supplementary Fig. 2c). For Dmd, a 32 nt miE modulates the structure of the highly conserved dystrophin C-terminus that interacts with other proteins^48^ (Supplementary Fig. 2d).

### Regulation of the ASD-risk gene splicing program during cortex development

**Figure 3:**
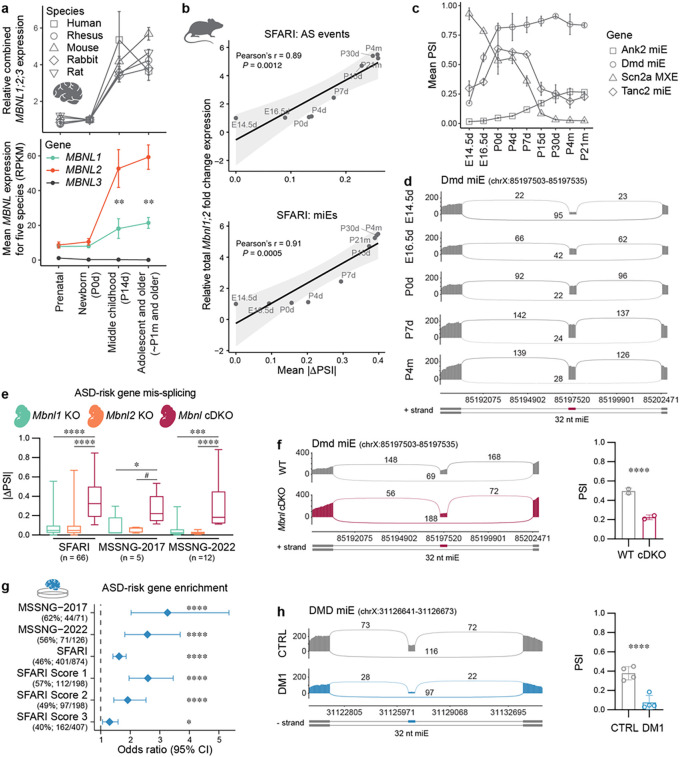
MBNL proteins governs developmental splicing transitions in ASD-risk genes. **a**, *MBNL1, MBNL2* and *MBNL3* gene expression levels in developing brains of five species. Top: total *MBNL* expression relative to newborn/postnatal day 0 (P0d) time point. Bottom: mean *MBNL* expression for five species at each developmental stage. Points show mean expression ± SD. Significant differences between MBNL1 and MBNL2 expression at different developmental stages were determined by a two-tailed t-test; ** *P* < 0.01. **b**, Correlation between *Mbnl* gene expression and mean |DPSI | values for MBNL-sensitive splicing changes in SFARI genes in developing WT mouse cortex. MBNL-sensitive AS events (top) and miEs only (bottom) were selected based on differential AS analysis in the *Mbnl* cDKO cortex. The Pearson correlation coefficient (r) and *P* values are shown. **c**, The line chart shows individual mean PSI values ± SD in the developing cortex for four MBNL-sensitive AS events in MSSNG-2017 and MSSNG-2022 genes at embryonic day 14.5 (E14.5d), E16.5, postnatal day 0 (P0d), P4d, P7d, P15d, P30d, postnatal month 4 (P4m), and P21m. **d**, Sashimi plots show Dmd miE splicing transitions during mouse cortical development (N = 2 for each time point). **e**, ASD-risk gene mis-splicing in *Mbnl1* KO (N = 2), Mbnl2 KO (N = 2) and *Mbnl* cDKO (N = 2) mouse E18.5 cortical neuron RNA-seq samples. The box plot shows the lower (25^th^ %ile), middle (median, 50^th^ %ile) and upper (75^th^ %ile) quartiles. Whiskers show minimum and maximum. Number of mis-spliced AS events are provided as n value. Statistical differences were determined by Kruskal-Wallis test followed by Dunn’s multiple comparison test: # *P* = 0.067, * *P* = 0.038, *** *P* = 0.0006, and **** *P* < 0.0001. **f**, Sashimi plot of embryonic *Mbnl* cDKO (N = 2) and WT (N = 2) RNA-seq samples for Dmd miE. The bar graph shows the mean PSI ± SD; **** FDR < 0.0001. **g**, MSSNG-2017, MSSNG-2022 and SFARI gene-set enrichment analysis for mis-spliced genes in 8-month-old DM1 brain organoid (N = 2 in 2 replicas). Points represent the OR and error bars represent the 95% CI. The vertical dashed line represents OR = 1; * FDR = 0.013 and **** FDR < 0.0001. **h**, Sashimi plot of DM1 (N = 2 in 2 replicas) and WT (N = 2 in 2 replicas) 8-month-old brain organoid RNA-seq samples for DMD miE. The bar graph shows the mean PSI ± SD; **** FDR < 0.0001.

To assess the developmental splicing pattern of ASD-risk genes, we analyzed gene expression data for five mammalian, including human, brains at different developmental stages^49^. Our analysis showed an evolutionarily conserved increase of *MBNL2* expression during neonate/P0 to middle childhood/P14 brain development (Fig. 3a and Supplementary Fig. 3a). Although *MBNL1* expression increases simultaneously, its expression in the developed brain is approximately 3-fold lower than *MBNL2*. To assess the association between *Mbnl1* and *Mbnl2* gene expression and MBNL-sensitive splicing transitions in the developing mouse cortex, we evaluated RNA-seq data from WT mice^50^. We computed mean |DPSI | values at nine developmental time points for AS events mis-spliced in ASD-risk genes in the *Mbnl* cDKO cortex and correlated them with *Mbnl* expression levels. As anticipated, the correlation between these variables was very strong for ASD-risk genes from the SFARI (*r* = 0.89, *P* = 1.2 × 10^−3^), MSSNG-2017 (*r* = 0.90, *P* = 9.0 × 10^−4^), and MSSNG-2022 (*r* = 0.91, *P* = 7.0 × 10^−4^) studies (Fig. 3b and Supplementary Fig. 3b). In agreement, the correlation remained strong for MBNL-sensitive miEs in the ASD-risk genes from the SFARI (*r* = 0.91, *P* = 5.0 × 10^−4^), MSSNG-2017 (*r* = 0.83, *P* = 5.5 × 10^−3^), and MSSNG-2022 (*r* = 0.76, *P* = 1.6 × 10^−3^) studies (Fig. 3b and Supplementary Fig. 3b). Differential AS analysis demonstrated that 48–56% of mis-spliced AS events in ASD-risk genes were significantly changed between embryonic and adult cortex (Supplementary Fig. 3c). For example, *Scn2a1* MXE, *Ank2* miE*, Tanc2* miE, and *Dmd* miE splicing transitions occurred at early developmental stages to reach a plateau postnatally between two and four weeks of age (Fig. 3c–d and Supplementary Fig. 3d), which is consistent with the developmental expression patterns of Mbnls (Fig. 3a).

To assess whether prenatal MBNL loss influences splicing of ASD-risk genes, we analyzed RNA-seq data of the primary embryonic cortical neuron samples from *Mbnl* cDKO, constitutive *Mbnl1*^−/−^ KO (hereafter *Mbnl1* KO), constitutive *Mbnl2*^−/−^ KO (hereafter *Mbnl2* KO) and WT mice ^51^. We performed differential splicing analysis followed by ASD-risk gene enrichment analysis. In agreement with the relatively low embryonic *Mbnl1* and *Mbnl2* expression levels (Fig. 3 and Supplementary Fig. 3a), we did not observe significant enrichment of mis-splicing for ASD-risk genes from the SFARI (OR = 1.3, FDR = 0.17), MSSNG-2017 (OR = 1.0, FDR = 1.00) and MSSNG-2022 (OR = 1.3, FDR = 0.62) studies in the embryonic *Mbnl* cDKO. However, in agreement with the additive effect of Mbnl paralogs loss, we noticed a greater degree of mis-splicing events in ASD-risk genes in *Mbnl* cDKO compared to *Mbnl1* KO and *Mbinl2 KO* embryonic cortical neurons, including *Dmd* miE (Fig. 3e–f and Supplementary Fig. 3e). To further investigate the impact of *DMPK* CTG^exp^ mutation on the ASD-risk gene splicing program in the developing human brain, we also analyzed DM1 and control brain organoid RNA-seq samples^52^ followed by the differential splicing and ASD-risk gene enrichment analyses. We found a significant enrichment of mis-spliced events in ASD-risk genes from the SFARI (OR = 1.6, FDR = 6.6 × 10^−11^), MSSNG-2017 (OR = 3.3, FDR = 1.3 × 10^−6^) and MSSNG-2022 (OR = 2.6, FDR = 3.6 × 10^−7^) studies in the DM1 brain organoid, including previously identified DMD miE (Fig. 3g–h). Overall, these results indicated that MBNL proteins govern the splicing patterns of multiple ASD-risk genes, including miEs, in the developing brain.

### MBNL2 loss causes ASD-risk gene mis-splicing in multiple brain regions

**Figure 4:**
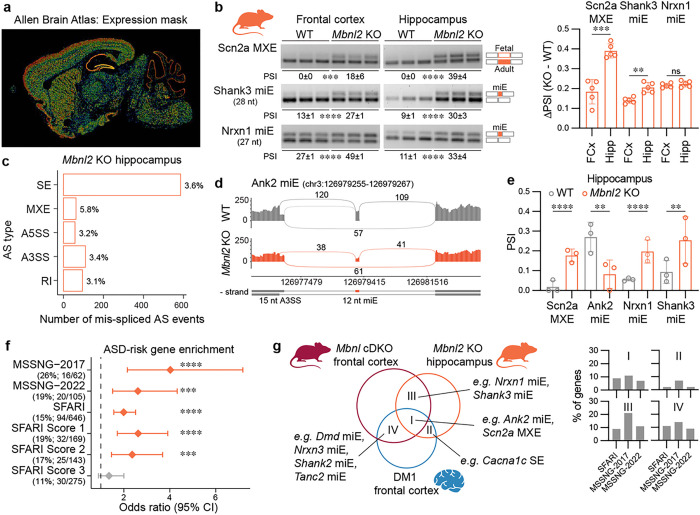
Mis-splicing in the *Mbnl2* knockout hippocampus. **a**, The Allen Mouse Brain Atlas (mouse.brain-map.org) shows the normalized color-coded Mbnl2 expression level (from blue-low to red-high) derived from the informatics data processing of *in situ* hybridization (ISH) results (mouse.brain-map.org/gene/show/69724)^75^. **b**, Representative RT-PCR splicing assay gels of Scn2a MXE, Nrxn1 miE and Shank3 miE in *Mbnl2* KO (N = 5) and littermate WT (N = 5) frontal cortex (FCx) and hippocampus (Hipp). Mean (D)PSI ± SD are shown below the gels or on a bar graph. Significant differences were determined by unpaired two-tailed t-test: ** *P* < 0.01, *** *P* < 0.001, **** *P* < 0.0001. **c**, Differential AS analysis in *Mbnl2* KO (N_XX_ = 3) compared to littermate WT control (N_XX_ = 3) hippocampus RNA-seq samples. The bar graph shows the number and percentage of significantly mis-spliced AS event types (|DPSI | > 0.1, FDR < 0.05). **d**, Ank2 miE sashimi plot of *Mbnl2* KO and WT hippocampus RNA-seq samples. **e**, Scn2a MXE, Ank2 miE, Nrxn1 miE, and Shank 3 miE mis-splicing in *Mbnl2* KO hippocampus RNA-seq. Bar graph shows the mean PSI ± SD; ** FDR < 0.01, **** FDR < 0.0001. **f**, MSSNG-2017, MSSNG-2022 and SFARI gene-set enrichment analysis for mis-spliced genes in *Mbnl2* KO hippocampus. Points represent the OR and error bars represent the 95% CI. The vertical dashed line represents OR = 1; *** FDR < 0.001, **** FDR < 0.0001 **g**, Venn diagram showing the overlap between mis-spliced ASD-risk genes in DM1 prefrontal cortex, *Mbnl* cDKO frontal cortex, and *Mbnl2* hippocampus RNA-seq samples. Bar graphs show the percentage of overlap for mis-spliced SFARI, MSSNG-2017 and MSSNG-2022 genes.

*Mbnl2* is the predominant gene paralog expressed in the adult human and mouse cerebral cortex, hippocampus, and cerebellum (Fig. 4a and Supplementary Fig. 4a-b), and these brain regions are known to be involved in ASD^53, 54^. To test the hypothesis that Mbnl2 loss perturbs splicing of ASD-risk genes in multiple brain regions, we performed RT-PCR splicing analysis of *Scn2a* MXE, *Nrxn1* miE and *Shank3* miE in frontal cortex, hippocampus, and cerebellum of adult *Mbnl2* KO and WT mice. Two of three tested AS events demonstrated the most profound mis-splicing in the hippocampus (Fig. 4b and Supplementary Fig. 4c-d). Thus, to investigate Mbnl2-mediated AS regulation in ASD-risk genes in the hippocampus, we performed differential splicing analysis on RNA-seq data from *Mbnl2* KO^55^. In total, 4% of AS events were perturbed in 8% of detected genes, including *Scn2a, Ank2, Nrxn1*, and *Shank3* (Fig. 4c–e and Supplementary Fig. 4e-f). As observed for the *Mbnl* cDKO, 53% of ASD-relevant gene lists were significantly enriched among the mis-spliced genes in the *Mbnl2* KO hippocampus, including ASD-risk genes from the SFARI (OR = 2.0, FDR = 7.2 × 10^−7^), MSSNG-2017 (OR = 4.0, FDR = 6.8 × 10^−^5) and MSSNG-2022 (OR = 2.6, FDR = 1.4 × 10^−3^) studies (Fig. 4f and Supplementary Fig. 4g). Approximately 9% of all mis-spliced ASD-risk genes in *Mbnl2* hippocampus overlapped with those found in DM1 prefrontal cortex and *Mbnl* cDKO frontal cortex (Fig. 4g). The most consistently mis-spliced events were *Ank2* miE and *Scn2a* MXE. Therefore, Mbnl2 loss alone impacts the alternative splicing of ASD-risk genes in multiple ASD-relevant brain regions, including the hippocampus.

### Direct regulation of ASD-relevant microexons by MBNL proteins

**Figure 5:**
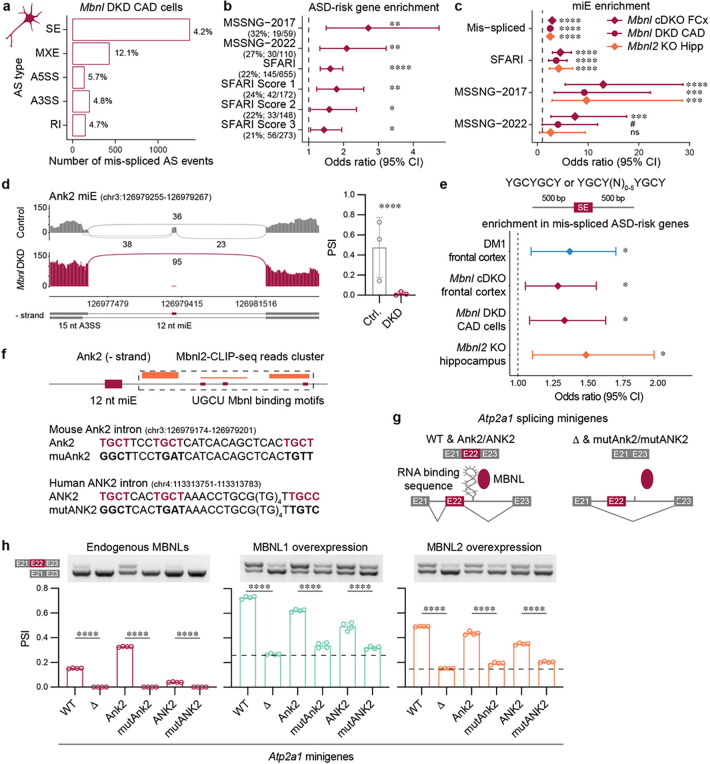
MBNL proteins directly regulate ASD-risk gene splicing. **a**, Differential AS analysis in *Mbnl* DKD (N = 3) compared to control (N = 3) CAD RNA-seq samples. The bar graph shows the number and percentage of significantly mis-spliced AS event types (|DPSI | > 0.1, FDR < 0.05). **b**, MSSNG-2017, MSSNG-2022 and SFARI gene-set enrichment analysis for mis-spliced genes in *Mbnl* DKD CAD cells. **c**, MiE enrichment analysis for SFARI, MSSNG-2017 and MSSNG-2022 mis-spliced SE events. **c,d**, Points represent the OR and error bars represent the 95% CI. The vertical dashed line represents OR = 1. **d**, Ank2 miE sashimi plot of *Mbnl* DKD (N = 3) and control (N = 3) CAD RNA-seq samples. Bar graph shows mean PSI ± SD. **e**, MBNL-binding motif enrichment near mis-spliced SE in ASD-risk genes. **f**, Mbnl2-CLIP-seq (N_XX_ = 3) reads cluster cover three UGCU motifs in intron downstream Ank2 miE. Mbnl binding sequences identified in mouse Ank2 and human ANK2 introns and their mutant variants used in heterologous *Atp2a1* splicing minigene experiments. **g**, Schematic of *Atp2a1* splicing minigene variants and regulation by MBNL proteins. *Atp2a1*-WT *Atp2a1*-*Ank2* (mouse), and *Atp2a1*-*ANK2* (human) contain functional MBNL-binding sequences and result in alternative E22 inclusion. In contrast, E22 exclusion occurs in *Atp2a1*-D, *Atp2a1*-mut*Ank2*, and *Atp2a1*-mut*ANK2* with deleted WT and inserted mutated mouse *Ank2* and human *ANK2* MBNL-binding sequences, respectively. **h**, *Atp2a1*-derived splicing minigenes regulation by endogenous MBNL, exogenous MBNL1 and MBNL2 proteins in HeLa cells (N = 4). Bar graphs show the mean Atp2a1 E22 PSI ± SD. Dashed line shows *Atp2a1*-D E22 inclusion as baseline for other splicing minigenes. Significant differences were determined by unpaired t-test: **** *P* < 0.0001. **b-e**, Points represent the OR and error bars represent the 95% CI. The vertical dashed line represents OR = 1; ns FDR = 0.11, # FDR = 0.052, * FDR < 0.05, ** FDR < 0.01. *** FDR < 0.001, **** FDR < 0.0001.

To support the observation that Mbnl proteins directly regulate splicing of high-confidence ASD-risk genes, we first performed differential AS analysis on RNA-seq data from a mouse brain-derived catecholaminergic (CAD) neuronal cell line with siRNA-mediated *Mbnl1*; *Mbnl2* double knockdown (hereafter *Mbnl* DKD) versus control^51^. In total, 5% of AS events in 15% of detected genes were mis-spliced (Fig. 5a and Supplementary Fig. 5a), and 45% of ASD-risk gene lists were significantly enriched among the mis-spliced genes in the *Mbnl* DKD CAD cell line, including ASD-risk genes from the SFARI (OR = 1.6, FDR = 2.2 × 10^−5^), MSSNG-2017 (OR = 2.7, FDR = 2.7 × 10^−3^) and MSSNG-2022 (OR = 2.1, FDR = 5.2 × 10^−3^) studies (Fig. 5b and Supplementary Fig. 5b). Like mouse *Mbnl* DKO frontal cortex and *Mbnl2* KO hippocampus, mis-spliced miEs in ASD-risk genes from the SFARI (OR = 3.7, FDR = 9.9 × 10^−6^), MSSNG-2017 (OR = 9.2, FDR = 2.1 × 10^−4^) and MSSNG-2022 (OR = 4.1, FDR = 5.2 × 10^−2^) studies were significantly enriched in *Mbnl* DKD CAD cells (Fig. 5c). We identified Ank2 miE mis-splicing similar to the previously analyzed DM1, *Mbnl* cDKO, and *Mbnl2* KO brain regions (Fig. 5d).

Next, we performed a MBNL binding site enrichment analysis for SE mis-splicing in ASD-relevant genes. Based on our previous MBNL-RNA interaction studies^17, 56, 57^, we determined YGCYGCY and YGCY(N)_0–5_YGCY as high affinity MBNL-binding sequences. We performed a genome-wide distribution analysis of intronic MBNL-binding motifs ±500 bp from alternative SE splice sites. We detected a significant enrichment of Mbnl binding sequences in mis-spliced ASD genes in DM1 (OR = 1.4, FDR = 0.011) and *Mbnl* cDKO (OR = 1.3, FDR = 0.012) frontal cortex, *Mbnl* DKD CAD cells (OR = 1.3, FDR = 0.011) as well as *Mbnl2* KO hippocampus (OR = 1.5, FDR = 0.011) (Fig. 5e).

Since our data indicated Mbnl proteins preferentially regulate ASD-risk gene miE splicing, we selected the highly conserved Ank212 nt miE, which was consistently mis-spliced in our various models, to study the molecular mechanism underlying miE splicing. To assess whether Mbnl directly regulates Ank2 miE inclusion, we analyzed Mbnl2 crosslinking and immunoprecipitation sequencing (CLIP-seq) samples from adult WT hippocampi^55^. A cluster of CLIP-seq reads indicates an Mbnl2-RNA interaction region. In agreement with previous studies demonstrating that Mbnl binding within the downstream intron of an alternative SE promotes its inclusion^55, 58^, we identified a Mbnl2-CLIP-seq cluster covering a conserved TGCT(N)3TGCT(N)_13–18_TGCT/C sequence ~55 bp downstream 5’ splice site in *Ank2/ANK2* intron (Fig. 5f and Supplementary Fig. 5c). Based on our previous MBNL-RNA interaction studies^17, 56, 57^, we anticipated this motif represents high affinity MBNL-binding sequences. *In silico* RNA secondary structure modeling predicted that MBNL binding motifs are localized in less structured RNA regions (Supplementary Fig. 5d). Since splicing of the *Ank2* miE to the A3SS results in mRNA isoforms differing by only 3 nt (as explained earlier), we took advantage of our previously developed *Atp2a1* E22 inclusion minigene (*Atp2a1*-WT) assay for RNA-MBNL interactions^56^. We deleted an experimentally confirmed MBNL-binding motif within the downstream intron 22 of the mouse *Atp2a1*-WT minigene (*Atp2a1*-D) and inserted the 90 bp mouse *Ank2* and human *ANK2* conserved intronic sequence TGCT(N)_3_TGCT(N)_13–18_TGCT/C (*Atp2a1-Ank2, Atp2a1-ANK2*) and mutated GGCT(N)_3_TGAT(N)_13–18_TGTT/C sequences (*Atp2a1*-mut*Ank2*, *Atp2a1*-mut*ANK2*;substitutions are underlined) (Fig. 5f–g). The disruption of YGCY (Y = U/C) motifs is known to lower the affinity of MBNL proteins for RNA^57^. We transfected HeLa cells with these *Atp2a1* minigenes and measured E22 inclusion by RT-PCR. In contrast to *Atp2a1*-D, *Atp2a1*-mut*Ank2*, and *Atp2a1*-mut*ANK2* minigenes, *Atp2a1*-WT, *Atp2a1*-Ank2, and *Atp2a1-ANK2* were sensitive to the endogenous level of MBNL proteins (Fig. 5h). To support our observation, we co-transfected *Atp2a1* minigenes and MBNL1, MBNL2 or EGFP (control) expression vectors. *Atp2a1-Ank2* E22 and *Atp2a1-ANK2* E22 were significantly more included than *Atp2a1*-mut*Ank2* and *Atp2a1*-mut*ANK2* (Fig. 5h and Supplementary Fig. 5e) indicating that MBNL proteins directly regulate miE splicing in an ASD-risk gene. All these results support our proposal that MBNL directly regulates the splicing of ASD-risk genes miEs.

### MBNL inhibition in DM1 mimics miE mis-splicing in idiopathic ASD

**Figure 6:**
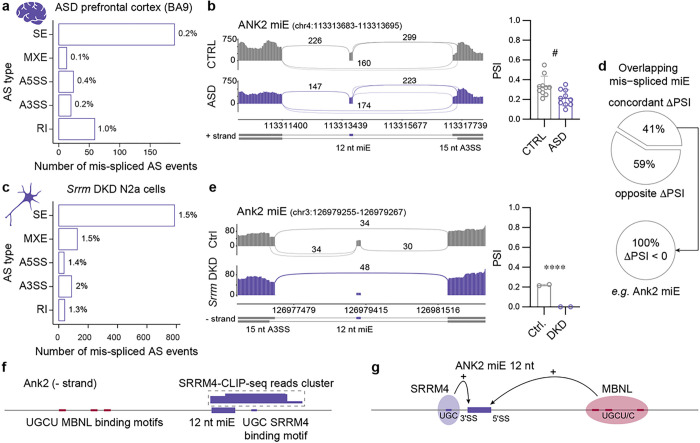
ANK2 microexon mis-splicing in ASD. **a**, Differential AS analysis in ASD (N = 10: N_XX_ = 1, N_XY_ = 9; sampling age: median = 51y, min = 38y, max = 67y; ASD confirmed by the Autism Diagnostic Interview-Revised (ADI-R; N = 8) or supported by records) compared to age-matched CTRL (N = 10: N_XX_ = 2, N_XY_ = 8; sampling age: median = 50y, min = 41y, max = 60y) frontal cortex (BA9) RNA-seq samples. AS mis-splicing criteria: |DPSI | > 0.1, FDR < 0.06. The bar graph shows the number and percentage of significantly mis-spliced AS event types. **b**, ANK2 miE mis-splicing in ASD. Sashimi plot of ASD (N = 8) and CTRL (N = 8) BA9 RNA-seq samples. The bar graph shows the mean PSI ± SD; # FDR = 0.056. **c**, Differential AS analysis in *Srrm* DKD (N = 2) compared to control (N = 2) N2a RNA-seq samples. Bar graph shows the number and percentage of significantly mis-spliced AS event types (|DPSI | > 0.1, FDR < 0.05). **d**, Top pie chart represents the percentage of mis-spliced miE that overlap between *Srrm* DKD N2a and *Mbnl* DKD CAD cells with concordant and opposite DPSI values. Bottom pie chart demonstrates all concordant miEs are excluded. **e**, Ank2 miE sashimi plot of *Srrm* DKD (N = 2) and control (N = 2) N2a RNA-seq samples. The bar graph shows mean PSI ± SD; **** FDR < 0.0001. **f**, SRRM4-CLIP-seq reads cover in Ank2 miE upstream intron containing UGC motif. **g**, SRRM4 and MBNL proteins promote ANK2 miE inclusion by binding to differently localized similar intronic sequence motifs.

To ascertain whether there were common mis-spliced genes and AS events between DM1 and ASD, we retrieved adult idiopathic ASD prefrontal cortex (BA9) samples from the PsychENCODE Consortium (Supplementary Table 1)^28, 59^. Our differential AS analysis revealed 0.3% of mis-spliced events in 2% of analyzed genes (Fig. 6a and Supplementary Fig. 6a), and 15 mis-spliced AS events overlapped between DM1 and ASD (OR = 3.5, *P* = 5.7 × 10^−5^, Fisher’s exact test), including ANK2 miE (Fig. 6b). Previous reports have linked neuronal miE mis-splicing in idiopathic ASD brains to reduced SRRM4 (nSR100) expression^30^. Like *Mbnl1* and *Mbnl2*, *Srrm3* and *Srrm4* are paralogs that regulate the same set of neuronal miEs^60^. To ascertain whether there are common mis-spliced ASD-relevant miEs between Mbnl loss and Srrm loss, we retrieved RNA-seq data from the mouse Neuro2a (N2a) cell line with siRNA-mediated *Srrm3*; *Srrm4* double knockdowns (hereafter *Srrm* DKD) versus control^60^. Our differential AS analysis revealed 2% of mis-spliced events in 7% of analyzed genes (Fig. 6c and Supplementary Fig. 6b), and 63% of ASD-risk gene lists were significantly enriched by the mis-spliced genes in the *Srrm* DKD N2a cell line (Supplementary Fig. 6c). As anticipated, *Srrm* DKD preferentially altered miE splicing (OR = 22, FDR 6.8 × 10^−207^), including miE in ASD-risk genes from the SFARI (OR = 23, FDR = 1.7 × 10^−34^), MSSNG-2017 (OR = 34, FDR = 5.1 × 10^−9^) and MSSNG-2022 (OR = 25, FDR = 1.9 × 10^−8^) studies (Supplementary Fig. 6d). In total, we identified a non-random overlap of 153 mis-spliced AS events between *Mbnl* DKD CAD and *Srrm* DKD N2a (OR = 4.5, *P* = 4.0 × 10^−44^, Fisher’s exact test), including 34 altered miE events (OR = 2.0, *P* = 4.5 × 10^−3^, Fisher’s exact test). 41% of overlapping mis-spliced miEs showed concordant DPSI changes in *Mbnl* DKD and *Srrm* DKD, all of which demonstrated exon exclusion in the transcripts (DPSI < 0) (Fig. 6d). For example, we identified the *Ank2* miE as an overlapping mis-spliced event that underwent exon exclusion in the transcripts in both *Mbnl* DKD and *Srrm* DKD cells (Fig. 5d and 6e).

SRRM4 protein promotes neuronal miE inclusion by binding to an intronic UGC motif approximately 15 nt upstream the 3′SS of targeted exon^61^. In contrast, MBNL proteins bind to downstream intronic UGCY motifs to promote alternative exon inclusion^58, 62^. To support that MBNL and SRRM4 regulate Ank2 miE inclusion binding to distinct sequences, we retrieved available CLIP-seq data from an N2a cell line expressing flagged SRRM4 protein^61^. As expected, we identified a SRRM4-CLIP-seq reads cluster covering a conserved UGC motif 9 nt upstream Ank2 miE, and there were no reads supporting SRRM4 interaction with the MBNL binding site and vice versa (Fig. 5f, 6f and Supplementary Fig. 6e).

In contrast to *MBNL2*, *SRRM4* has a relatively higher expression in embryonic compared to postnatal brain in human and mouse (Supplementary Fig. 6e). As predicted, *Srrm4* and *Srrm3* gene expression levels were unchanged in *Mbnl* cDKO frontal cortex, *Mbnl2* KO hippocampus, and *Mbnl* DKD CAD cells (Supplementary Fig. 6f). Interestingly, we noticed the significant 28% reduction of *SRRM4* RNA in DM1 brain, however this downregulation did not correlate with CTG^exp^ (*r* = −0.41, *P* = 0.36) (Supplementary Fig. 6f-g). These results indicate that the MBNL and SRRM proteins regulate splicing of ASD-relevant miEs, such as *ANK2* miE (Fig. 6g), in an independent manner.

### Social interaction deficits in *Mbnl2* knockout and *Dmpk* 3’UTR CTG^exp^ knockin mice.

**Figure 7:**
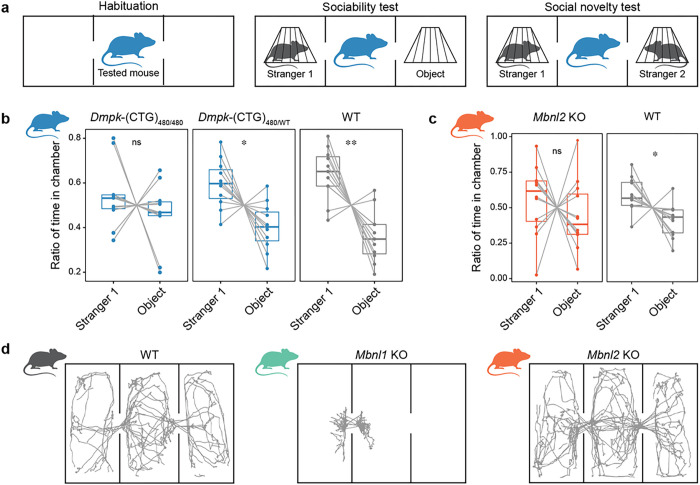
ASD-associated social deficit in two DM1 mouse models. **a**, Scheme of the three-chamber sociability and social novelty test. Each experiment consisted of the following 10 min consecutive phases: habituation, sociability, and social novelty. **b,c**, The ratio of time in the chamber with novel animal (Stranger 1) and object during the sociability test. Note that time spent in the middle chamber is not included. **b**, WT mice (N = 11: N_XX_ = 7, N_XY_ = 4), heterozygous *Dmpk*-(CTG)_480/WT_ (N = 11: N_XX_ = 7, N_XY_ = 4), and homozygous *Dmpk*-(CTG)_480/480_ (N = 11: N_XX_ = 7, N_XY_ = 4). The box plot shows the lower (25^th^ %ile), middle (median, 50^th^ %ile) and upper (75^th^ %ile) quartiles. Whiskers show minimum and maximum. Paired dots represent all sample data points, including outliers. Statistical differences were determined by the paired t-test: ns *P* = 0.38 (t = 0.9134, df = 10), * *P* = 0.013 (t = 2.997, df = 10), ** *P* = 0.0025 (t = 4.012, df = 10). **c**, WT mice (N = 12: N_XX_ = 9, N_XY_ = 3), Mbnl2 KO (N = 12: N_XX_ = 9, N_XY_ = 3). The box plot shows the lower (25^th^ %ile), middle (median, 50^th^ %ile) and upper (75^th^ %ile) quartiles. Whiskers show minimum and maximum. Paired dots represent all sample data points, including outliers. Statistical differences were determined by the paired t-test: ns *P* = 0.43 (t = 0.8193, df = 11), * *P* = 0.023 (t = 2.641, df = 11). **d**, Assessment of mouse movement activity.

Ekström and colleagues have reported that DM1 children have a higher incidence of impaired social interaction and communication skills^63^, and thus we tested sociability in our DM1 mouse models using the three-chamber test. The three-chamber test involves three phases: habituation, sociability, and social novelty^64^ (Fig. 7a). We first selected heterozygous *Dmpk* 3’UTR (CTG)_480/WT_ knockin (hereafter *Dmpk*-(CTG)_480/WT_ KI) and homozygous *Dmpk*-(CTG)_480/480_ KI mouse models to study phenotypic outcomes. Both *Dmpk*-(CTG)_480/WT_ KI and *Dmpk*-(CTG)_480/480_ KI reproduce characteristic DM1 pathological molecular signatures, including MBNL sequestration on *Dmpk* 3’UTR (CUG)_480_ RNAs, RNA mis-splicing in the vulnerable cell types, and DMPK protein loss^65^. Importantly, the molecular phenotypes are significantly more exaggerated in homozygous *Dmpk*-(CTG)_480/480_KIcompared to heterozygous *Dmpk*-(CTG)_480/WT_ KI^65^. In the sociability phase of the three-chamber test, WT and heterozygous *Dmpk*-(CTG)_480/WT_ KI mice spent significantly more time in the chamber with a novel animal (Stranger 1) than a novel object (Fig. 7b). In contrast, homozygous *Dmpk*-(CTG)_480/480_ KI mice showed no significant preference for the chamber with novel animal over the novel object (Fig. 7b), signifying a lack of sociability.

To test the hypothesis that MBNL inhibition underlies the social deficit, we evaluated *Mbnl2* KO and *Mbnl1* KO mouse models. The *Mbnl1* KO is characterized by muscle (*e.g*., myotonia), immune system and vision pathology^66, 67^, whereas the *Mbnl2* KO exhibits central nervous system abnormalities, including neuronal morphology and synaptic changes^45, 55, 68^. Like homozygous *Dmpk*-(CTG)_480/480_KI, and in contrast to WT, *Mbnl2* KO mice did not spend significantly more time in the chamber with a novel animal (Fig. 7c). Additionally, *Mbnl2* KO mice also showed no significant preference for social novelty when presented with a familiar animal (Stranger 1) and a novel animal (Stranger 2) in the social novelty phase (Supplementary Fig. 7a). Since *Mbnl1* is the dominant *Mbnl* paralog expressed in skeletal muscles, testing *Mbnl1* KO mice in the social test failed to provide reliable results due to their profoundly limited mobility evident during the habituation phase (Fig. 7d). In contrast, *Mbnl2* KO mice did not exhibit significant exploratory locomotor deficits in the three-chamber test and the open-field test (Fig. 7d and Supplementary Fig. 7b).

These mouse behavioral results showed that either *Dmpk*-(CTG)*480/480* expression or MBNL2 protein loss led to social interaction deficits, a key diagnostic feature of DM1-associated ASD. The variability observed in the three-chamber test for both homozygous *Dmpk*-(CTG)_480/480_ and *Mbnl2* KO mice suggests incomplete penetrance of this phenotype.

## Discussion

Here, we delineate the mechanisms underlying a specific ASD-linked tandem repeat expansion and its phenotypic consequences. We provide evidence that *DMPK* 3’UTR CTG^exp^ and its subsequent inhibition of MBNL’s RNA splicing activity adversely impacts the developmental ASD-risk gene splicing program, which leads to social interaction deficits, as we demonstrated in the mouse models. Thus, we propose that ASD can arise from a gene-specific tandem repeat expansion through an RNA-mediated gain-of-function mechanism whereby symptoms are a consequence of altered RNA splicing of multiple ASD-risk genes during brain development.

Aberrant RNA splicing is a characteristic feature of the ASD brain, including neuronal miE mis-splicing shown in approximately one-third of ASD cases^27, 30^. Although miEs are regulated by multiple RNA-binding proteins, their abnormal exclusion in ASD brains has been linked to downregulated *SRRM4* expression. For example, the *ANK2* miE 12 nt analyzed in this study is commonly mis-spliced in both DM1 and ASD brains and is co-regulated by MBNL and SRRM4 proteins. Like MBNL inhibition, SRRM4 haploinsufficiency not only causes miE mis-splicing, but also a social deficit in mice^33^. Additionally, AS events in the DM1 brain can mimic ASD-associated variants. For example, *SCN2A* MXE mis-splicing results in a protein isoform differing by a single negatively charged amino acid (adult-to-fetal: D209N) in the extracellular loop of the Na_v_1.2 channel voltage-sensing domain (Supplementary Fig. 6d). Previous research has demonstrated that similar to the ‘fetal’ MXE inclusion, ASD-associated *SCN2A* variants reduce neuronal excitability^69–71^. The role of mis-splicing in DM1-associated ASD is additionally supported by recent clinical trial results for tideglusib (AMO-02). ASD symptoms were improved in some of the treated children with DM1^72^. In preclinical studies tideglusib, a small-molecule inhibitor of glycogen synthase kinase 3 (GSK3), reduces CUG^exp^ RNA levels and corrects aberrant splicing in DM1-derived cells and two DM1 repeat expansion mouse models^73^.

Studies on tandem repeat expansions provide a unique opportunity to investigate the mechanistic threads in ASD, as was successfully demonstrated for the prototypical example of the CGG expansion in the *FMR1* 5’ untranslated region (5’UTR). The *FMR1* 5’UTR CGG^exp^ underlies Fragile X-Associated Disorders, including Fragile X Syndrome (FXS) which is the most common monogenic disorder comorbid with ASD^74^. Here, we provide a molecular mechanism for the *DMPK* 3’UTR CTG^exp^ as a second example of a tandem repeat expansion leading to ASD traits.

## Methods

### Mouse models

All relevant ethical regulations for animal testing and research were observed, and this study received approval from the University of Florida Institutional Animal Care and Use Committee (IACUC). All animal procedures and endpoints were in accordance with IACUC guidelines and animals were sacrificed in accordance with IACUC-approved protocols. B6.129S1-*Mbnl1*^*DE3*/DE3^ (*Mbnl1* KO) and B6.129S1-*Mbnl2*^*DE2*/DE2^ (*Mbnl2* KO) have been described^55, 66^. B6-*Dmpk*-(CTG)_480_ (^³^N6) line was derived from the previously described FVB-*Dmpk*-(CTG)_480_^65^. We used Mbnl2 KO (N = 12: N_XX_ = 9, N_XY_ = 3) and WT littermate mice (N = 12: N_XX_ = 9, N_XY_ = 3) as well as heterozygous *Dmpk*-(CTG)_480/WT_ (N = 11: N_XX_ = 7, N_XY_ = 4), homozygous *Dmpk*-(CTG)_480/480_ (N = 11: N_XX_ = 7, N_XY_ = 4), and WT littermate mice (N = 11: N_XX_ = 7, N_XY_ = 4). All behavioral analyses were performed between 8 weeks and 6 months of age followed by brain harvesting. Mice were housed under specific pathogen-free conditions. Both the humidity (50%–70%) and temperature (70–75°F) were controlled, and the room was maintained on a 12:12 light:dark cycle (lights off at 8:00 pm). Mice were ear-notched, and tail-snipped for identification and genotyping. Same-sex littermates were group-caged (2–4 mice/cage) at weaning in cages with water and standard rodent chow available *ad-lib*. The mice remained in the same cage group throughout the behavioral experiments.

### Three Chamber Test

Three chamber test was used to assess sociability in mouse models. The rectangular three-chambered apparatus consisted of three 20 cm × 40.5 cm × 22 cm chambers separated by clear Plexiglass walls. The walls had small doors that could be lifted or closed between phases to allow chamber access or prevent it. Throughout the test, the center chamber remained empty, and objects or target (Stranger) mice were placed in the left or right chambers. The test mouse was the mouse that had its behavior analyzed. The target mice (Strangers 1 and 2) were matched in both age and sex to the test mouse and were placed into the test to provide a social stimulus. The test mice did not undergo any other experiments prior to being placed in the three-chambered social test. Similarly, the target mice only were subject to being novel mice in the three-chambered test and were not involved in any other experiments. Wire cups were used to confine the target mice while allowing for social investigation by the test mouse. Before beginning, the two target mice were habituated for ten minutes in the inverted wire cups that they were subsequently placed in during the social test. During this habituation, we observed if target mice exhibited aggression or abnormal behaviors, such as excessive grooming, bar-biting, and jumping, that could interfere with the test and provided grounds for their exclusion. None of the target mice used in this study met these criteria for exclusion.

The habituation phase for the test mouse followed the habituation of the target mice. All chambers were completely empty during this phase. This phase allowed the test mouse to acclimate to the chambers and allowed us to assess if they showed a preference for one side before any novel objects or animals had been placed in the chamber.

For the sociability phase, an empty inverted wire cup was placed in one chamber while an inverted wire cup with one of the target animals (Stranger 1) was placed in the chamber on the opposite side. The chamber that contained the target animal alternated with each animal that was being tested. The test mouse was placed in the center chamber, and once the doors were lifted, left to explore all chambers for ten minutes. The test animal was allowed to interact with the cup with or without a social partner present for ten minutes.

For the social novelty phase, the same target animal (Stranger 1) that was used in the sociability phase remained in its place and the previously empty wire cup became occupied by a novel target mouse (Stranger 2). The test mouse was placed in the center chamber to begin and allowed to explore all chambers for ten minutes once the doors were lifted. This phase assessed whether the animal displayed more investigative behavior towards the novel target mouse (Stranger 2) or displayed a preference for the familiar mouse (Stranger 1).

After the three phases of the social test were completed and the animals were placed back in their home cages, the interior of the chambers and the wire cups were sanitized with ethanol before proceeding with another test mouse. Illumination was kept even on both sides of the apparatus. The test was conducted in a quiet room with minimal visual distractions and was recorded overhead using a video camera. Each video specified the date, test animal ID, and target mice used.

Mouse video tracking during habituation phase was performed using ToxTrac (v 2.98)^76^.The recorded videos were observationally coded by human raters using Behavioral Observation Research Interactive System (BORIS v 8.1.2) software^77^. Time in each chamber and the number of social/object interactions were coded during the sociability and social novelty phases, respectively. Social interactions were operationally defined as the test mouse sniffing the target mouse, which could include nose-to-nose interaction, the test mouse sniffing any other part of the body of the target mouse, or nose-to-cup interaction, and rearing on the wire cup with the target mouse^64^. Object interactions only applied to the sociability phase and were defined as sniffing or rearing on the wire cup that did not have a target mouse in it. During the social novelty phase, social interactions were coded for both chambers, differentiating which animal was the novel one and which was the familiar one. Twenty per cent of the coded observations were randomly selected and independently scored by another researcher to determine the agreement between raters. A criterion of 85% or greater inter-observer agreement was established. If the behavioral scores were recorded between 1 second of each other for point events and, for durations of behavior, were 2 seconds of the start and stop time, it was counted as a scoring agreement. These parameters were set to account for the reaction time of the scorers. All the data included in this study met the criteria for 85% inter-rater agreement.

### Automated Open Field Test

Mice were acclimated to the procedure room for approximately two hours before the test. For the open field, test mice were then placed in the center of the darkened activity-monitoring 17” × 17” chamber (Med Associates), and mouse movement was traced for 30 min. Analysis was performed with Activity Monitor (MED Associates, Inc.) software. For the final statistical analysis, only 5–30 min interval was taken.

### Cell Line

HeLa cells were cultured in were grown in Dulbecco’s Modified Eagle Medium (DMEM; high-glucose, GlutaMAX supplement, pyruvate) supplemented with 10% fetal bovine serum and 1x antibiotic and antimycotic (All Thermo Fisher Scientific) at 37°C with 5% CO_2_.

### Minigenes

EGFP-MBNL1-41, EGFP-MBNL2-38, Atp2a1-WT and Atp2a1-D minigenes were previously described^17, 57^. Mouse Ank2 and mutAnk2 as well as human ANK2 and mutANK2 splicing minigenes were generated by cloning DNA oligonucleotides between *Not*I and *Sal*I restriction sites in Atp2a1-D minigene. 120 bp DNA oligonucleotides contained selected TGCT(N)_3_TGCT(N)_13–18_TGCT/C or mutated GGCT(N)_3_TGAT(N)_13–18_TGTC sequences (substitutions are underlined) as well as *Not*I and *Sal*I restriction sites at 5’ and 3’ ends respectively. DNA oligonucleotide sequences are listed in the key resources table. The complementary single-stranded DNA oligonucleotides (100 μM) were annealed in Annealing Buffer (10 mM Tris, pH 7.5, 50 mM NaCl, 1 mM EDTA) at 95°C for 5 minutes followed by cooling to 25°C for 45 minutes. Annealed oligonucleotides were digested with *Not*I and *Sal*I restriction enzymes (New England Biolabs), purified using Clean-Up Concentrator Kit (A&A Biotechnology), and ligated. The design of the hybrid *Atp2a1* minigenes preserves RNA structures within a thermodynamically stable region at the Atp2a1 insertion site^57^. Final splicing minigenes were tested by Sanger sequencing.

### Transfection

HeLa cells were seeded on 12-well plates filled with 1 mL of medium and allowed to grow up to 50–60% of confluence priori transfection. Cells were transfected with Lipofectamine 3000 (Invitrogen) according to the manufacturer’s protocol. For exogenous splicing analysis, HeLa cells were co-transfected with 200 ng of the indicated minigene construct and 500 ng of the EGFP-MBNL1-41, EGFP-MBNL2-38 or EGFP expressing vector. The cells were harvested 48 hours after transfection.

### RNA Isolation

Mouse tissues were homogenized in TRIzol (Ambion) with 1.5 mm zirconium beads in a Bead Ruptor 12 (OMNI International). Total RNA from mouse tissues and HeLa cells were isolated by using TRIzol Reagent (Invitrogen)/TRI Reagent (Sigma-Aldrich) and the Direct-zol RNA MiniPrep Kit (Zymo Research)/Total RNA Zol-Out D Kit (A&A Biotechnology) with on-column DNase digestion according to the manufacturer’s protocol.

### RT-PCR Splicing Analysis

Total RNA (1–2 μg) was reverse transcribed using the GoScript Reverse Transcription System (Promega)/High-Capacity cDNA Reverse Transcription Kit (Thermo Fisher Scientific) with Random Primers (Promega, Thermo Fisher Scientific) according to the manufacturer’s protocol. PCR was conducted using GoTaq G2 Flexi DNA Polymerase (Promega). PCR products were resolved on 2% agarose gels stained with ethidium bromide and gels visualized on a Molecular Imager ChemiDoc XRS + (BioRad)/G:Box (Syngene) and analyzed using Image Lab (BioRad)/GeneTools software (Syngene). All primers and PCR product sizes are listed in the key resources table.

### RNA-seq and CLIP-seq Analysis

All RNA-seq and CLIP-seq data accession numbers are listed in the key resources table. Reads were aligned to the human hg38 or mouse mm10 genomes using STAR (v 2.7.5c)^78^. Splicing analysis was performed using rMATS (v 4.1.0)^79^. Sashimi plots were generated using ggsashimi.py script^80^. Median coverage was used to generate the plot (-A median). The total numbers of junction reads are showed. The introns were compressed for better representation (--shrink). Transcript expression quantification was performed using Salmon (v 1.1)^81^, and differential gene expression analysis was performed using DESeq2 (v 1.32.C)^82^.

### ASD-risk Gene Datasets

See Supplementary Table 2.

### Gene Expression Database

Evo-devo mammalian organs (apps.kaessmannlab.org/evodevoapp/)^49^. dbGaP accession number phs000424.v8.p2 (www.gtexportal.org/home/datasets).

### RNA Structure Prediction

RNAfold (rna.tbi.univie.ac.at).

### Protein Structure Prediction

The modeled structures of mouse proteins up to 50 aa or 214 aa for SHANK3 were predicted using the UCSC ChimeraX AlphaFold tool with the use of ColabFold, an optimized version of AlphaFold2 with default parameters^83, 84^. Protein fragments used for structure modeling with miE-encoded residues are underlined.

Shank3 without miE-encoded sequence: GGLGSLLDPAKKSPIAAARLFSSLGELSTISAQRSPGGPGGGASYSVRPSGRYPVARRAPSPVKPASLERVEGLGAGVGGAGRPFGLTPPTILKSSSLSIPHEPKEVRFVVRSVSARSRSPSPSPLPSPSPGSGPSAGPRRPFQQKPLQLWSKFDVGDWLESIHLGEHRDRFEDHEIEGAHLPALTKEDFVELGVTRVGHRMNIERALRQLDGS

Shank3 with miE-encoded sequence: GGLGSLLDPAKKSPIAAARCAVVPSAGCALQQPR

Nrxn1 without miE-encoded sequence: RLPDLISDALFCNGQIERGCEGPSTTCQEDSCSNQGVCLQQ

Nrxn1 with miE-encoded sequence: RLPDLISDALFCNGQIERGCEVALMKADLQGPSTTCQEDSCSNQGVCLQQ

Dmd without miE-encoded sequence: TGLEEVMEQLNNSFPSSRGHNVGSLFHMADDLGRAMESLVSVMTDEEGAE

Dmd sith miE-encoded sequence: TGLEEVMEQLNNSFPSSRGRNAPGKPMREDTM

### Post-translational Modifications

PhosphoSitePlus (v 6.7.1.1; www.phosphosite.org).

### Allen Mouse Brain Atlas

Mouse Brain Atlas (mouse.brain-map.org). Experiments were performed on P56d old male C57BL/6J mice.For a detailed description of *in situ* hybridization (ISH) procedure and informatics data processing see: help.brain-map.org/display/mousebrain/Documentation.

### Group Size

Group size determinations were based on assuming power = 0.8, α = 0.05 with effect sizes estimated based on our previous studies using G*Power (v 3.1) software. RNA-seq maximum group sizes and sample characteristics were predetermined. We analyzed sex- and age-matched groups.

### Statistical Analysis

Whole transcriptome statistical analysis for splicing and gene expression was performed using rMATS (v 4.1.0)^79^ and DESeq2 (v 1.32.C)^82^, respectively. The odds ratio (OR) was calculated using ‘epitools’ package in R, and the statistical significance was determined based on Fisher’s exact test followed by the multiple comparison correction using the FDR method. Other statistical analyses were performed using GraphPad Prism (v 9.5.1). The normal distribution was assessed by the Shapiro–Wilk test followed by parametric or nonparametric tests and the post hoc test for multiple comparisons. Graphs were generated in R using the ‘ggplot2’ package and GraphPad Prism (v 9.5.1) software. Details are specified in the figure legends.
